# Does Trapping Influence Decision-Making under Ambiguity in White-Lipped Peccary (*Tayassu pecari*)?

**DOI:** 10.1371/journal.pone.0127868

**Published:** 2015-06-10

**Authors:** Selene Siqueira da Cunha Nogueira, Iurianny Karla Fernandes, Thaise Silva Oliveira Costa, Sérgio Luiz Gama Nogueira-Filho, Michael Mendl

**Affiliations:** 1 Universidade Estadual de Santa Cruz, Ilhéus, Bahia, Brazil; 2 University of Bristol, Langford, Bristol, United Kingdom; Université Pierre et Marie Curie, FRANCE

## Abstract

The white-lipped peccary (*Tayassu pecari*) is an endangered species whose bold anti-predator behaviour in comparison to related species may increase its vulnerability to hunting and predation. We used a judgement bias test to investigate whether captive peccaries that had recently experienced a trapping event made more ‘pessimistic’ decisions under ambiguity. If so, this would indicate (i) that the procedure may induce a negative affective state and hence have welfare implications, and (ii) that the species is able to adopt a cautious response style despite its bold phenotype. Eight individuals were trained to ‘go’ to a baited food bowl when a positive auditory cue (whistle; CS+) was given and to ‘no-go’ when a negative cue (horn A; CS-) was sounded to avoid a loud sound and empty food bowl. An ‘ambiguous’ auditory cue (bell; CSA) was presented to probe decision-making under ambiguity. Individuals were subjected to three tests in the order: T1 (control-no trap), T2 (24h after-trap procedure), and T3 (control-no trap). In each test, each animal was exposed to 10 judgement bias trials of each of the three cue types: CS+,CS-,CS_A_. We recorded whether animals reached the food bowl within 60s (‘go’ response) and their response speed (m/s). The animals varied in their responses to the CS_A_ cue depending on test type. In all tests, animals made more ‘go’ responses to CS+ than CS_A_. During control tests (T1 and T3), the peccaries showed higher proportions of ‘go’ responses to CS_A_ than to CS-. In T2, however, the animals showed similar proportions of ‘go’ responses to CS_A_ and CS-, treating the ambiguous cue similarly to the negative cue. There were differences in their response speed according to cue type: peccaries were faster to respond to CS+ than to CS- and CS_A_. Trapping thus appeared to cause a ‘pessimistic’ judgement bias in peccaries, which may reflect a negative affective state with implications for the welfare and management of captive individuals, and also function to increase caution and survival chances following such an event in the wild environment.

## Introduction

Cognitive processes such as the ability to appraise and judge stimuli and events may influence and be influenced by affective states [1], [2], [3]. Empirical and theoretical findings suggest that animals (including humans) in a negative affective state should be more likely to judge ambiguous cues as if they predict a negative event—a ‘pessimistic’ response—than animals in a positive state [2], [4]. There is growing support for these predictions (e.g. [5]) indicating that such ‘cognitive biases’ may be useful new indicators of emotional state in animals. Most research to date has been on domestic animals and, where wild animals have been studied, manipulations of affective state, if used at all, have involved husbandry procedures such as provision or removal of enrichment (starlings: [6], [7]; bears: [8] or veterinary inspection (rhesus monkeys: [9]. Here, using white-lipped peccary (*Tayassu pecari*), we investigated whether trapping, which simulates some aspects of hunting (being stressful and involving capture) and occurs during both management of captive animals and also in the wild, leads to ‘pessimistic’ judgements under ambiguity indicative of a negative affective state.

In addition to assessing whether the trapping methods used here generate a negative affective state and hence may have welfare implications for the management of captive peccaries, we were also interested in how peccaries adjusted decision-making following such events. Predominantly frugivorous, the white-lipped peccary depends on palm trees, distributed in huge clusters in the forest, to obtain food [10]. When fruit is lacking, herds expand their home range [11] or adopt nomadic-like behavior [12] searching for food resources in new areas. The white-lipped peccary’s anti-predator strategy is somewhat unusual, involving mass attack of the predator, apparently in response to behaviour of the dominant group leader [13]. Such bold exploratory and anti-predator behavioural traits may not to be adaptive in high-risk areas. Sih et al. [14] suggest that it is more adaptive to be shy (or ‘pessimistic’ in our case) in high-risk environments than to be bold. The bold trait of white-lipped peccary when facing predators and its vulnerable conservation status may thus be associated.

The white-lipped peccary is categorized as vunerable by IUCN [15] and the population is declining in numbers. Studies have recorded areas with local extinction due to over-hunting and habitat fragmentation [16], [17], and the species appears to be more susceptible to environmental impact than the related collared peccary, *Pecari tajacu* [18]. The collared peccary is more wary of predators and shows an escape as opposed to a confrontational response, and it is possible that the white-lipped peccary’s bold behavioural style may be a contributory factor to its vulnerability. For example, its anti-predator strategy is successful with unarmed human beings, but if hunters are carrying guns, its cohesive and confrontational behaviour and apparent dependence on the actions of the dominant animal can lead the entire group towards their death [19], [20], [21]. The same kind of decision situation might occur in different species in different contexts and, depending on the species anti-predator strategy, a more fixed or flexible response may influence its chances of survival. We were therefore also interested in whether white-lipped peccary can modify their decision-making and implement a more cautious or ‘pessimistic’ response style following a trapping event, or whether they persist with bold and ‘optimistic’ choices even after experiencing danger.

## Materials and Methods

### Ethics statement

This work followed the “Principles of laboratory animal care” (NIH publication No. 86–23, revised 1985) and was approved by the Committee of Ethics for Animal Use (CEUA) at the Universidade Estadual de Santa Cruz (protocol #021/13).

### Animals and facilities

**Fig 1:**
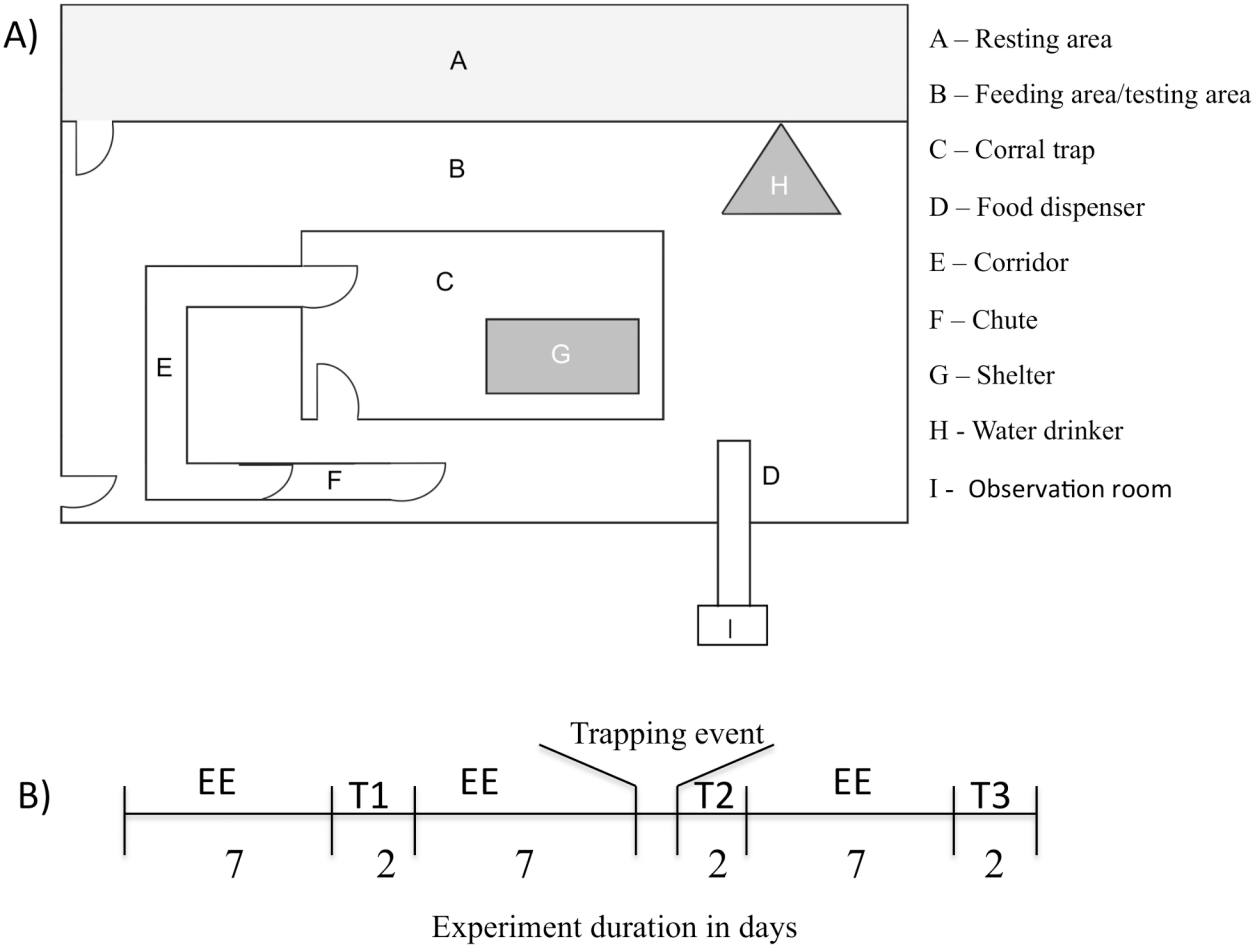
(A) Schematic drawing of the area used and (B) the experiment timeline in days at LABET. Symbols correspondence: EE- environment enrichment; T-test; M-capture, handling event.

We carried out an experiment with eight adult white-lipped peccaries (four males and four females) at the Applied Ethology Laboratory, Universidade Estadual de Santa Cruz, Brazil (14°47’39.8”S, 39°10’27.7”W). The experimental animals (approximately four years old) were from the same herd (N = 18), born and raised in captive conditions. Four adult animals from this herd were taken out of the study because they did not learn the cue commands (see details in training session). In addition, we did not include siblings or juveniles, as our focus was not on ontogenetic differences. The entire social group was kept in a semi-natural wire-fenced (1.5m height) area of 940m^2^. Part of this area was designated to animal feeding (376m^2^) and the rest (564m^2^) was for animal resting or refuge (Fig 1A). The resting area was composed of natural tropical vegetation, while the feeding area had a dirt-floor without vegetation. Each area (feeding and resting) had one water drinker (0.7m long X 0.55m wide X 0.18m height) where animals could access the water *ad libitum*.

The feeding area contained a corral-trap and a chute (trap and capture facility, respectively, Fig 1A). The corral-trap (10.0m long X 93.0m wide) was used as a bait station to attract the herd. The chute (1.78m long X 0.70m wide X 1.0m high, Fig 1A), allowed us to individually capture the experimental animals, simulating hunting—a stressful event [22]. The corral-trap walls (1.5m height) were of wire fencing and covered with a black plastic material to block the animals’ view of the external environment. The chute was connected to the corral-trap by a corridor (6.5m long X 0.70m wide X 1.0m high; Fig 1A), allowing us to select which individual from the corral-trap would be captured in the chute when necessary.

During all experiments the animals were maintained in a spatial and temporal feeding enrichment schedule proposed by Nogueira et al. [23] to keep animals exploring, simulating the usual foraging behaviour of wild peccaries [24], and minimising inactivity due to lack of stimulation. In this regime, food was provided twice at random times between 0800 and 1700 hours in feeders chosen randomly each time [23]. The animals’ general diet was composed of a mixture of maize grain, soybean meal, and mineral salts, providing 12% crude protein and 3,300 Kcal/kg of gross energy (700g/animal) [25], except during the reward training and test days (see details below) when the animals were positively rewarded with slices of cassava roots (*Manihot* sp.), their favourite food (Nogueira-Filho *pers*. *com*.). The animals’ training and judgement bias tests were carried out in the feeding area, outside the trap and capture facility (Fig 1A) as follows.

### Animal training

After many unsuccessful attempts at individual training, we trained the animals together and then individually tested them in a judgement-bias experiment (see below). The individual training may have failed because of white-lipped peccaries’ close herding tendencies [13] and divergent individual responses to new situations when isolated from the dominant animal. In addition, it is important to highlight that although these animals live in captivity they are not tame, and they can express wild and aggressive behaviour towards human beings, making handling difficult.

To train the animals, we set up a food dispenser in the feeding area, made of a PVC tube (6.0m long X 0.15m diameter). This food dispenser was connected to the researchers’ observation room (Fig 1A). One end, located inside the feeding area, was 1.0m above the food bowl, and the other was raised up and placed inside the observation room so that one person could deliver the food (positive reinforcement, S+) through the dispenser at the exact moment that the animal arrived at the dispenser after the observer provided an auditory signal. Douglas et al. [26] found it difficult to train domestic pigs to discriminate a graded series of tones within a limited time window, and due to the constraints and challenges of working with a wild species and the lack of availability of a tone generator at the study site, we also used distinct auditory stimuli (whistle, horns, bell) as cues which we assumed could be learnt rapidly in a species with good hearing [13].

Following the Harding et al. [1] paradigm, animals were trained by operant conditioning [27] to ‘go’ to a baited food bowl when they heard one auditory cue (whistle; the positive conditioned stimulus: CS+), and to ‘no-go’ when a different auditory cue (horn A; CS-) was sounded in order to avoid a loud sound (horn B) and empty food bowl. We measured all sound frequencies using a decibel meter (Digital Minipa MSL-1352C, São Paulo, Brazil).

### Training animals to respond to the ‘go’ (CS+) and ‘no-go’ (CS-) cues

We started with ‘go’ (whistle to CS+; 85 Db at source; mean frequency of 3,281 Hz) training and then moved on to ‘no-go’ (horn A to CS-; 74 Db at source; mean frequency of 281 Hz) training after animals learned the first positive cues. Thus, during the training sessions the animals were in the resting area before the training trial started. The animal-keeper attracted them from the resting area to wait in front of the gate between the resting and feeding area (Fig 1A) by using a voice command. The trial started when the animals were waiting for the cue command close by the gate. At the first auditory cue (CS+) command, the animal-keeper opened the gate to release the herd to get access to the food bowl located in the feeding area (Fig 1A). Each correct ‘go’ response to the CS+ (moving to the food bowl) was rewarded with ~100g of cassava root slices released from the food dispenser, whilst a ‘no-go’ response to the CS+ was not rewarded or punished. We tried to guarantee that all individuals were rewarded adding more cassava slices if higher-ranking individuals appeared to be monopolising the food. During the first two days of training sessions for CS+ we repeated the whistle cue for a sequence of four whistle bout (~ 4 sec each) with ~25 seconds intervals between bouts, totalling 90 seconds until the animals turned in the direction of the sound source and obtained the food. After these two days we decreased the number of whistling bouts to one, and the animals learned to come to the bowl to get food after one signal.

To train animals to respond to the ‘no-go’ cue, we used almost the same procedure. The animals started in the resting area and the animal-keeper attracted them to wait for the cue command at the gate. The trial started when we sounded a horn A (CS-) and the animal-keeper opened the gate. The animals were then able to access the feeding area but had to stay at least 2m away from the food bowl (the 2m distance was indicated to the researcher by ink marks on the ground) to avoid a loud noise. Each correct ‘no-go’ response to the CS- was not rewarded or punished, but each incorrect ‘go’ response (go to food bowl) was punished by three bouts (~ 4 sec each) of bursts of horn B noise (95 Db at source; mean frequency of 469 Hz), with ~5 seconds intervals between bouts. We tested many sounds as potential aversive stimuli before the study began, and chose this sound because the animals clearly avoided it. All sound cues were emitted from the observer room close to the food dispenser.

We performed two CS+ training trials per day for 11 consecutive days, totalling 22 trials; and also performed two CS- training trials per day for just four consecutive days, because animals learned the ‘no-go’ response to the CS- faster than the ‘go’ response to the CS+. A learning criterion of at least 70% correct responses in all training trials (CS+ and CS-) was used. As mentioned earlier, four animals failed to achieve this and were not included in the testing sessions.

### Trapping event

Following training, judgement bias tests were carried out as described below. The trap, capture, and handling procedures were performed 24 hours before the second judgement bias test (T2) (Fig 1B). After the animal-keeper attracted the white-lipped peccaries to the feeding area by using a voice command, three people carried out the trapping event by using nets to drive animals to the corral-trap—a procedure lasting an average of 6 min—and then locking them in the trap. One by one, the keeper then moved the corralled animals to the corridor and captured each one in the chute for 3 minutes. After this time, the keeper opened the chute gate and moved the individual to a restraining cage (0.90m long X 0.52m wide X 0.58m high). When the animal was caught in this cage, the keeper and a veterinarian weighed and handled the white-lipped peccary for medical proposes (parasites or skin injuries), both procedures lasting 3 minutes. The complete procedure of trapping, capture, and handling took about 30 minutes in total. This procedure induces a physiological stress response in white-lipped peccary, causing an increase of up to 800% in glucocorticoid levels [22]. After these procedures each individual was released to the resting area. Thus, all animals were trapped on the same day and time and then tested at T2 during the following 24 hours.

### The judgement-bias test procedure

In judgement bias tests, an ‘ambiguous’ (CS_A_) auditory cue (bell: 76Db at source; mean frequency of 2,813Hz) was presented to investigate whether animals responded to this cue as if predicting the reward (‘optimistic’ response) or punishment (‘pessimistic’ response) [1]. Individuals were subjected to three tests in the order: T1 (control-no trap), T2 (24hrs after trap/capture/handling procedures) and T3 (control-no trap) (Fig 1B). The first test (T1) was carried out seven days after animals had started exposure to the enrichment regime (see Animals and facilities), which they then continued to experience throughout the rest of the study (Fig 1B). Test T2 was carried out 8 days after T1, and animals experienced the trapping event on the day prior to testing. T3 followed seven days after T2 (see Fig 1B). The three tests plus the day of trapping event spanned 28 days. During the periods before T1 and T3, the peccaries were not trapped or handled, and we avoided human contact or disturbance as far as possible.

Judgement bias trials ran from 0700 until 0930 in the morning and from 1530 until 1800 in the afternoon. We tested all eight white-lipped peccaries per day, four in the morning and four in the afternoon. During each of the three tests (T1,T2,T3), each individual completed a block of five trials per cue type per day over two consecutive days, completing 30 judgement bias trials (10 of each of the three cue types (CS+,CS-,CS_A_) per animal, per test (i.e. 90 trials in total across tests T1–T3). We waited for up to 60s for the individuals’ responses to each cue type, with intervals of 60s between cues, 2min intervals between different cue types, and 2min intervals between individuals.

To start the judgement bias trial, the animal-keeper attracted the herd from the resting area, as described for training commands and lured them to the corral-trap in order to identify four peccaries to be tested in that data collection session. For practical reasons and to minimise interference with the animals, we chose the closest animals to the test area for individual testing. The animal-keeper released the first animal from the corral to be tested in to the feeding area just after the first cue command was sounded. After release the test animal had no visual contact with the others inside the corral (see Fig 1A). The order of the testing trials (CS+, CS- or CS_A_) was randomly determined. We recorded whether the animal reached the food bowl (‘go’ response) within 60s, and its speed (distance travelled (m) / time (s)) to get the reward of 20g of cassava in the food bowl, or whether it remained at least 2m (‘no go’ response) from the bowl for the whole of the 60s. After the animal had consumed the reward, or 60s had elapsed following a ‘no-go’ response, the keeper attracted the animal back to the corral-trap door by using a voice command. We then sounded the next trial (CS+, CS- or CS_A_; randomly determined) and repeated this procedure until each cue had been sounded five times. We then released the test animal in to the resting area and, after an interval of 2 min, carried out the same procedure with the next peccary. We recorded the animals’ responses by using a digital camcorder (JVC GZ-HD500; Tokyo, Japan).

### Statistical Analyses

We calculated the proportion of trials on which each animal reached the food bowl within 60s (‘go’ response) for each cue type, and their speed to get to the bowl. If the animal reached a different point of the feeding area after being released from the corral, and stopped before getting to the food dispenser, we measured the distance between this first stopping point and the food dispenser. We analysed data using a GLM with repeated measures followed by *post hoc* Tukey test. Test type (T1, T2, T3) and cue type (CS+,CS-, CS_A_) were within-subjects factors and we examined their effects, including interactions, on the proportion of ‘go’ responses made. We analysed the mean response speed data using the same statistical model. Data fulfilled parametric requirements of normality of residuals and homogeneity of variance and are presented as mean ± S.D. All analyzes were performed using Statistica version 7.0—StatSoft, Tulsa, OK, USA, with significance level *P* < 0.05.

## Results

### Comparison of ‘go’ responses in the three tests (T1, T2, and T3)

**Fig 2:**
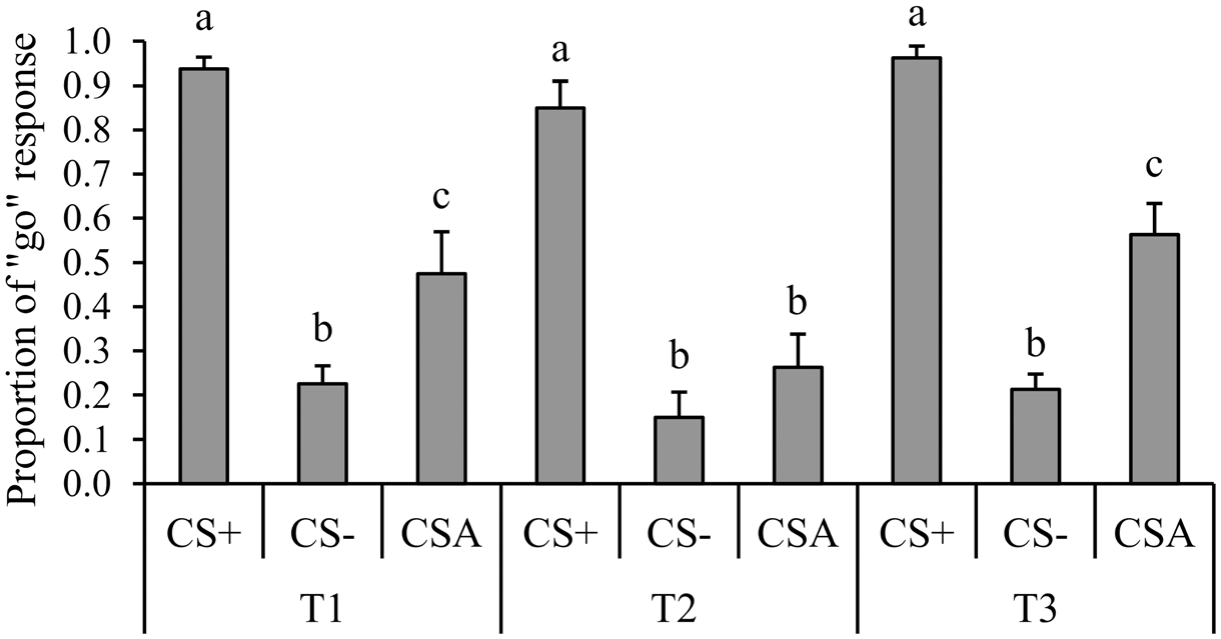
The average (+ SE) proportion of ‘go’ responses of white-lipped peccaries in each test (T1, T2 and T3) by each cue type (CS+, CS-, CS_A_). Different letters (a≠b≠c) represent significant differences and same letters indicate non-significant differences.

We found an interaction (*F*_4, 28_ = 3.79, *P* = 0.01) between test type (T1, T2, T3) and cue type (CS+, CS-, CS_A_). *Post hoc* test revealed that the white-lipped peccaries clearly discriminated (*P*s < 0.0001) the CS+ (0.97 ± 0.12), and CS- (0.18 ± 0.12) cues in all tests, which represented efficient learning of both sound stimuli (CS+ and CS-). However, the peccaries varied in their responses to ambiguous (CS_A_) cues depending on the test type (Fig 2). During the first control test (T1), *post hoc* test showed that the animals exhibited a higher (*P* = 0.03) mean proportion of ‘go’ responses to the ambiguous cue (CS_A_ = 0.48 ± 0.14) than to the negative cue (CS- = 0.23 ± 0.12). In T2 (after trapping-handling), the peccaries showed no difference (*P* = 0.74) in the mean proportion of ‘go’ responses made to the ambiguous cue (CS_A_ = 0.25 ± 0.21) and negative cue (CS- = 0.15 ± 0.16). In the second control test (T3), peccaries showed a higher (*P* = 0.0002) proportion of ‘go’ responses after the ambiguous cue (CS_A_ = 0.56 ± 0.20) than after the negative cue (CS- = 0.21 ± 0.10).

### Comparison of response speed in tests (T1, T2, and T3)

**Fig 3:**
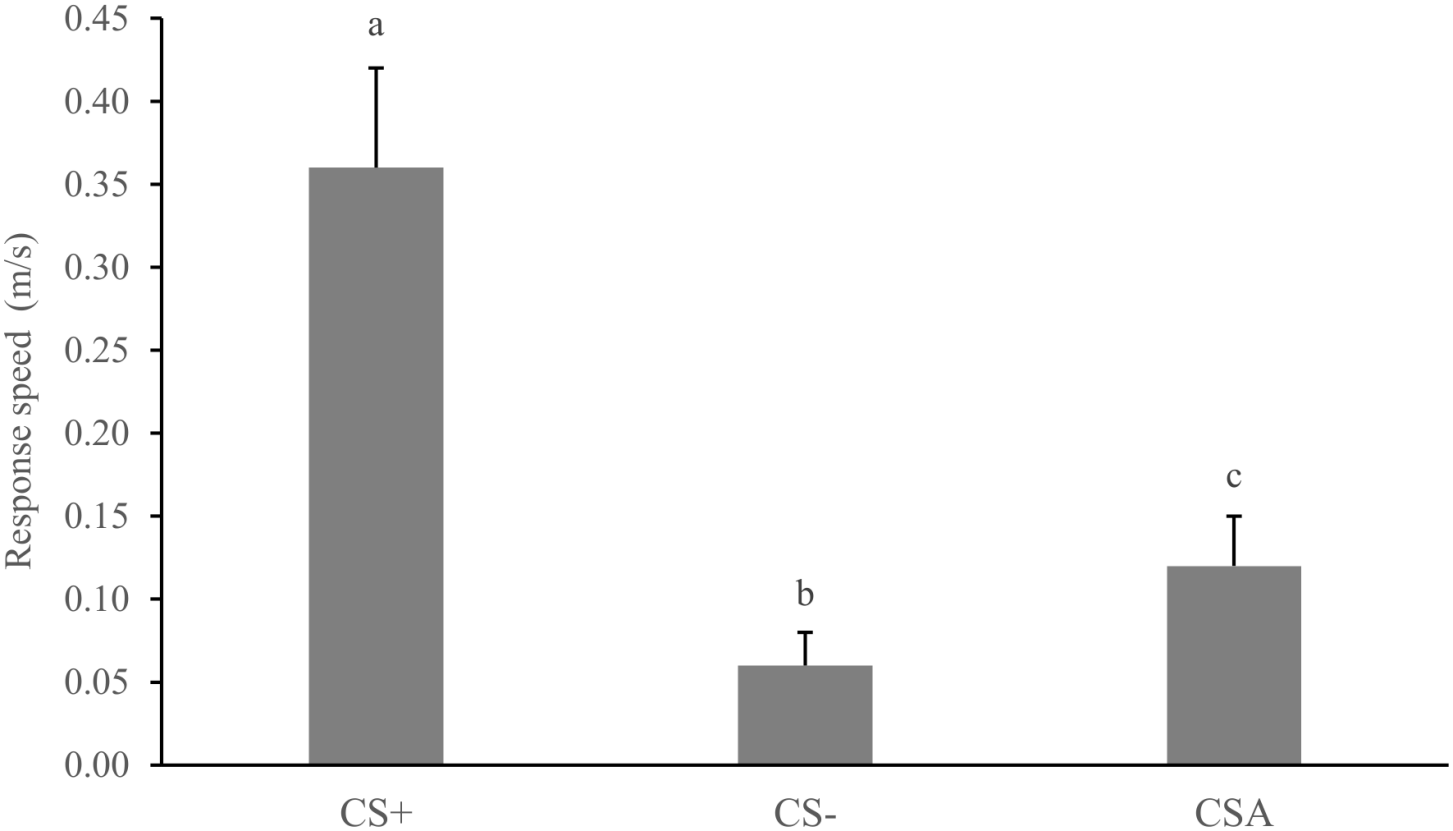
The average (+ SE) speed of responses (m/s) of white-lipped peccaries approaching the feed dispenser according to cue type (CS+, CS-, CS_A_). Different letters (a≠b) represent significant differences and same letters indicate non-significant differences.

The animals’ means response speed did not differ between the three tests (T1 = 0.21 ± 0.12, T2 = 0.16 ± 0.13, and T3 = 0.17 ± 0.12 m/s; *F*_2, 14_ = 1.20, *P* = 0.33). The animals, however, showed differences in response speed according to cue types (CS+, CS-, CS_A_) (*F*_2, 14_ = 39.54, *P* = 0.00001). The *post hoc* test showed that the peccaries showed the highest mean response speed (*P*s < 0.0002) to the positive cue (CS+ = 0.34 ± 0.14 m/s) relative to the negative (CS- = 0.06 ± 0.07 m/s), and ambiguous (CS_A_ = 0.12 ± 0.10 m/s) ones (Fig 3).

## Discussion

This study indicates that the cognitive bias paradigm could detect a potential affective state impact of a simulated hunting event (trapping and handling) on decision-making under ambiguity in white-lipped peccaries. Peccaries made ‘intermediate’ judgements of ambiguous cues under control conditions, but showed a relatively negative or ‘pessimistic’ judgement of the ambiguous signal during the 24 hours following the hunting simulation event, which may have increased caution after this event. Other studies using the cognitive bias paradigm have also demonstrated a negative judgement of ambiguity under assumed stressful or challenging conditions (e.g. unpredictable housing changes: [1]; predator presence/cues: [28]; physical restraint: [29]), indicating that changes in decision-making under ambiguity could be a useful indicator of affective state [5], and hence that trapping may induce a negative state in peccaries.

### The use of the judgement-bias paradigm in peccaries

The mean response speed to the CS+ cues was faster than to the CS- and CS_A_ cues across all three test types. Furthermore, even when animals made a ‘go’ response to CS- or CS_A_ cues, we informally observed that they hesitated in their approach to the food dispenser, something which did not happen in response to CS+ cues. This indicates that animals learnt the discrimination task, and that the food (cassava roots) worked as a reward for peccaries. In addition, the proportions of ‘go’ responses to CS+ and ‘no go’ responses to CS- were not affected by test type indicating that the peccaries maintained a similar motivation to get food and to avoid noise in response to the trained cues, even following the adverse event in Test 2. Thus, it was only responses to ambiguity that were affected by simulated hunting, as would be predicted if the degree of uncertainty associated with CS+ and CS- cues is low [4]. Similar results have been observed in other species (e.g. *Rattus norvegicus*: [30],[31]; rhesus macaques:[9]).

The intermediate proportions of ‘go’ responses to ambiguous stimuli (CS_A_) when compared to responses to positive (CS+) and negative stimuli (CS-) shown under control conditions suggests that the cue was treated as providing uncertain information as to whether to perform the ‘go’ or ‘no-go’ response. This is a typical response to ambiguity seen in many judgement bias studies where cues are presented on a simple unidimensional scale (e.g. sound cues differing only in tone frequency (e.g. [1], [31])). In our study, although cues did not vary along a unidimensional scale for various practical reasons, the ‘intermediate’ responses to ambiguity reflect those seen in other studies, and are also very similar to the findings of [26] who worked with domestic pigs, and also used sound cues that differed in more than one perceptual dimension. It is of interest that apparently categorically different sound stimuli are treated in this way in the judgement bias test. They may be quicker to train and their use thus requires further investigation, although they will not be able to provide ordinal generalisation curve data that can be generated using graded ambiguous stimuli on a unidimensional scale (e.g. ambiguous cues of 3 different frequencies between single-frequency training cues).

The peccaries’ responses to the ambiguous cue in the second baseline control test (T3) were similar to (slightly higher than) those seen in the initial baseline control test (T1), indicating that the animals did not learn across trials that this cue was not rewarded, as has been suggested in some other studies (e.g. [32], [33], [34]. Consequently, the increased ‘no-go’ responding to the ambiguous cue in the test following the adverse event (T2) is unlikely to have been due to extinction of response to this cue, but rather due to an affect-related bias in judgement of ambiguity.

### The possible influence of cognitive bias on white-lipped peccary survival

If our results reflect changes in decision-making that would occur in response to a hunting / predation event in the wild, we can speculate on their consequences for peccary survival. The white-lipped peccary’s cohesive behaviour promotes an opportunity for hunters to kill many individuals in a single hunting event [13]. If the dominant animal gets shot, the herd members appear to become disorientated, and an easy target for hunters [19], [20], [35] or other predators. In this context, pessimistic or shy behaviour by dominant peccaries during and following a predator attack may enhance survival chances by motivating escape as opposed to confrontational behaviour.

If only the more ‘pessimistic’ or shy individuals escape from hunting events, whilst bolder animals are shot, hunting may act as a selection pressure for those individuals who are more ready to demonstrate a ‘pessimistic’ / shy response to challenge. This may influence species exploratory and anti-predator behavioural traits. ‘Pessimistic’ individuals would be expected to avoid novel environments and ambiguous stimuli [4], behaviour which, in the case of the white-lipped peccary, could jeopardize populations during seasons when food is scarce. At these times, peccary need to search for food resources in new areas and expand their home range [11] or adopt nomadic-like behaviour [12]. Hunting-driven selection for shy / ‘pessimistic’ individuals may affect such foraging behaviour, compromising the peccary’s survival when food is limited.

Our results revealed that eight days after trapping/capture (T3-control), the peccaries’ response to ambiguity had reverted to one similar to that displayed during the initial baseline control test (T1). Enrichment provided to the animals in this study, previously tested as positive for peccaries [23], may have facilitated such resilience. Bethell et al. [9] studying rhesus monkeys and Douglas et al. [26] studying pigs also observed that animals’ baseline responses to ambiguity can be re-established after an environmental event, perhaps reflecting an underlying resilience to long-term changes in affective state following relatively short-lived challenges. Nevertheless, the effects of continuous negative environmental events such as high hunting rates in the Amazon forest, may be an important indirect factor contributing to the decrease in body size [36], [37]) and demographic changes [38] that have been reported for white-lipped peccary. Further studies are required to investigate this hypothesis.

The hunting simulation (trapping and capture) is a stressful event for animals [39] and may have deleterious effects on affective processes and cognitive abilities [40]. Encounters with hunters in the wild or other disturbances such as habitat fragmentation are likely perceived by animals as negative and the present study suggests that such events may lead to changes in decision-making that could have implications for survival in nature. Although we know little about many behavioural characteristics of white-lipped peccaries in their natural environment [12], it is likely that cognitive strategies used in response to environmental challenges [41], and during food, water and refuge acquisition, will influence survival [20]. Further research is required to determine whether environmental changes, such as hunting pressure and habitat disturbance, affect cognitive function in ways which do actually alter chances of survival, particularly if they decrease survival and hence favour the extinction process.

In summary, our study indicates that adverse events can lead to a more cautious ‘pessimistic’ response style in white-lipped peccaries, potentially mediated by a negative affective state, which may have adaptive value [4] depending on the environmental and temporal context as previously discussed. Affect-induced cognitive biases may therefore have implications for the survival of wild and endangered species, and the interplay between stress, negative affect and decision-making requires further investigation [40].
